# Supplementary material for: Predicting visual function by interpreting a neuronal wiring diagram
Source: Nature. 2024 Oct 2;634(8032):113–23. doi: 10.1038/s41586-024-07953-5 (PMC11446822; doi:10.1038/s41586-024-07953-5)
Supplement: Supplementary file 6 — CRF and ERF predictions for individual TmY4 and TmY9 cells. Analogous to Supplementary Data 3, but for TmY target types. Shown are the top four monosynaptic pathways, the strongest pathway passing through each of the top ten intermediary types (ranking from Extended Data Fig. 7), and the trisynaptic pathway Tm1–TmY–Dm3–TmY (see the section entitled Prediction of spatial normalization). [file 41586_2024_7953_MOESM6_ESM.zip › DataS4/TmY9q/720575940643607604.pdf]

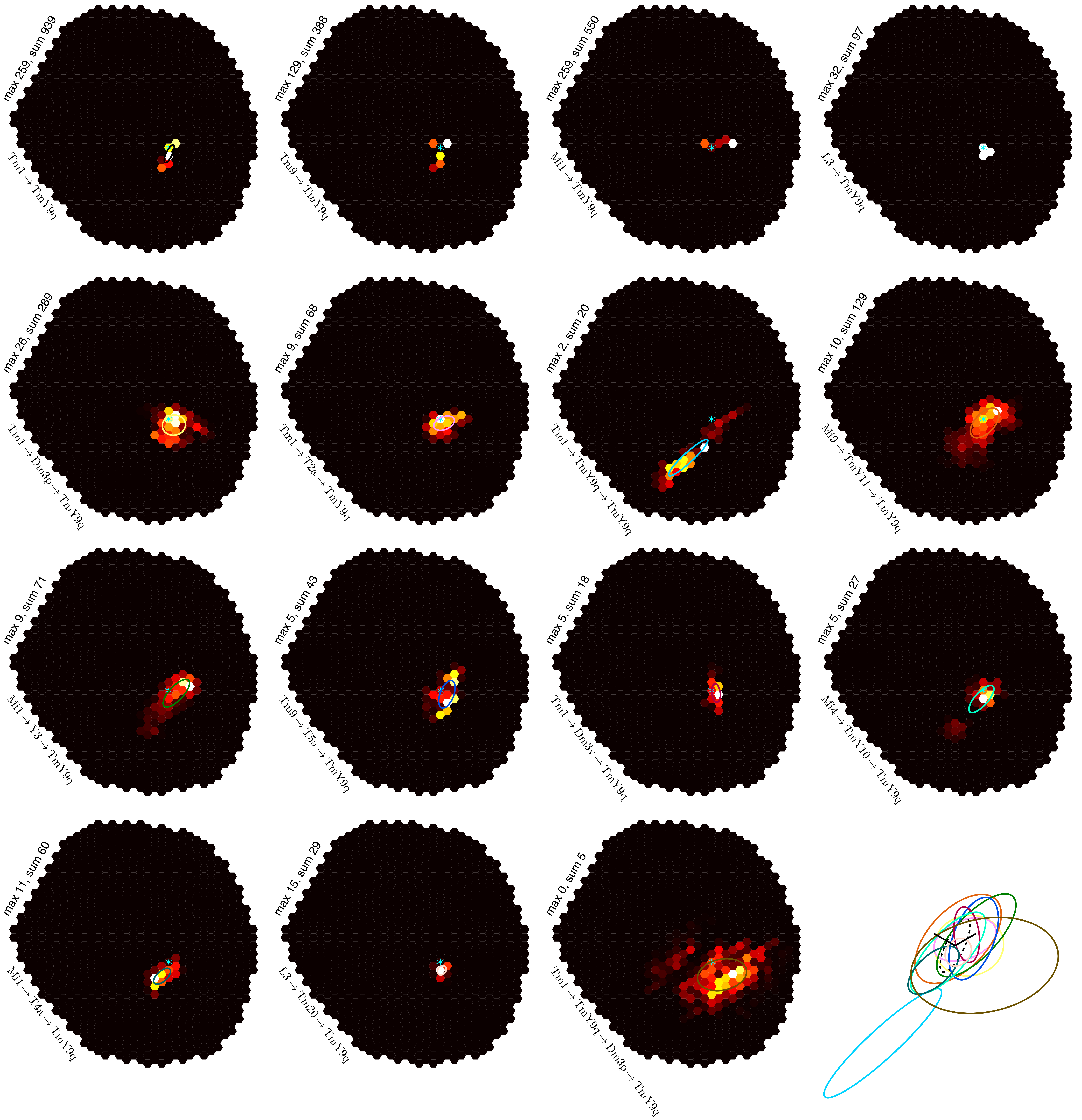
max 259, sum 939
Tm1 → TmY9q
max 129, sum 388
Tm9 → TmY9q
max 259, sum 550
Mi1 → TmY9q
max 32, sum 97
L3 → TmY9q
max 26, sum 289
Tm1 → Dm3p → TmY9q
max 9, sum 68
Tm1 → T2a → TmY9q
max 2, sum 20
Tm1 → TmY9q → TmY9q
max 10, sum 129
Mi9 → TmY11 → TmY9q
max 9, sum 71
Mi1 → Y3 → TmY9q
max 5, sum 43
Tm9 → T5a → TmY9q
max 5, sum 18
Tm1 → Dm3v → TmY9q
max 5, sum 27
Mi4 → TmY10 → TmY9q
max 11, sum 60
Mi1 → T4a → TmY9q
max 15, sum 29
L3 → Tm20 → TmY9q
max 0, sum 5
Tm1 → TmY9q → Dm3p → TmY9q
